# Cardioprotective Potential of Garlic Oil and Its Active Constituent, Diallyl Disulphide, in Presence of Carvedilol during Chronic Isoprenaline Injection-Mediated Myocardial Necrosis in Rats

**DOI:** 10.3390/molecules26175137

**Published:** 2021-08-25

**Authors:** Syed Mohammed Basheeruddin Asdaq, Abdulhakeem S. Alamri, Walaa F. Alsanie, Majid Alhomrani

**Affiliations:** 1 Department of Pharmacy Practice, College of Pharmacy, AlMaarefa University, Dariyah, Riyadh 13713, Saudi Arabia; 2 Department of Clinical Laboratory Sciences, The Faculty of Applied Medical Sciences, Taif University, Taif 21944, Saudi Arabia; a.alamri@tu.edu.sa (A.S.A.); w.alsanie@tu.edu.sa (W.F.A.); m.alhomrani@tu.edu.sa (M.A.); 3 Centre of Biomedical Sciences Research (CBSR), Deanship of Scientific Research, Taif University, Taif 21944, Saudi Arabia

**Keywords:** SOD, catalase, garlic oil, diallyl disulfide, carvedilol, cardiac troponin C, IL-1β, TNF-α, IL-6, NF-b p65

## Abstract

In isoprenaline (ISO)-induced myocardial infarcted rats, garlic oil (GO) and its main ingredient, diallyl disulfide (DADS), were examined for cardioprotective effects when used with carvedilol (CAR). GO, DADS and CAR were given to rats in their respective groups, either alone or together, with the addition of isoprenaline (3 mg/kg/day, subcutaneously) during the last 10 days of treatment. At the end of 14 days of treatment, blood samples were collected, the hearts were excised under anesthesia and weighed. Heart tissue homogenate was used to measure superoxide dismutase (SOD), catalase (CAT), and thiobarbituric acid reactive substances (TBARS). Furthermore, the serum activities of cardiac markers, including lactate dehydrogenase, creatine kinase, and cardiac troponin, were checked. Moreover, inflammatory markers including tumor necrosis factor alpha, interleukin one beta, interleukin six, and kappa bp65 subunit were assessed. Rats that received GO, DADS, and CAR exhibited a significant increase in the cardiac antioxidant enzyme activities with a simultaneous decrease in serum cardiac markers enzymes and inflammatory markers. The TBARS were significantly reduced in rats that received treatment. The addition of carvedilol to GO or DADS significantly elevated antioxidant activities and decreased the release of cardiac enzymes into blood circulation. Both DADS and GOl were almost similar in efficacy, indicating the potential role of DADS in garlic oil-mediated cardioprotection. Combining GO or DADS with CAR increased CAR’s cardioprotective impact and protected rats from developing ISO-induced myocardial infarction.

## 1. Introduction

The use of herbal remedies or nutritional supplements in conjunction with modern medicine is widespread all over the world. They are used with the expectation that adding herbs or nutrients to a prescription regimen would bring additional benefits and/or reduce drug side effects [1]. The data on the impact of concurrent administration of herbs or nutrients with conventional drugs are evolving but not available for most of the commonly used combinations. Nevertheless, there is published literature that clearly demonstrates the interference of herbs and conventional drugs in the pharmacokinetic and pharmacodynamic profiles of each other [2].

One of the most dreadful diseases is myocardial infarction (MI), also known as a heart attack (major ischemic heart disease). MI is expected to kill over 23.3 million people by 2030, according to estimates. In Asian countries such as India and Malaysia, mortality is rising at an alarming rate [3,4]. The most common cause of MI is atherosclerosis, which causes an imbalance in supply and demand in the myocardium, resulting in hypoxia and cellular waste accumulation that may result in the death of myocytes due to ischemia-induced free radical generation [5]. Despite this, the specific pathophysiology of MI is still unknown. However, inflammation and necrosis have been shown as major contributors to MI in a number of research studies [6,7].

Isoprenaline (ISO) is a nonselective catecholamine/beta-adrenergic receptor agonist. Since the chronic administration of ISO causes myocardial ischemia, hypoxia, and necrosis, with a fall in myocardial compliance, it is one of the most common models used in determining the cardioprotective efficacy of novel drugs and also to study the pathological consequences of human myocardial impairments [8]. Chronic ISO treatment (3 mg/kg/day) causes a rise in reactive oxygen species (ROS) in cardiac hypertrophy, which can cause changes in wall stiffness and impact cardiac function by causing ventricular hypertrophy and heart failure [9].

Garlic bulbs, which come from the plant *Allium sativum* (family Amaryllidaceae), are a common flavoring ingredient in a range of recipes. Garlic is also well-known for its therapeutic benefits. The garlic bulb and its numerous preparations, such as garlic oil, garlic powder, and various garlic extracts, are referenced in various traditional medical systems for their therapeutic effects [10]. Garlic’s remarkable biological effects include oxidative radical scavenging potential, cardioprotective abilities, in addition to being a successful adjuvant in managing several types of cancers, which have been proven in various research studies in recent decades [11,12,13,14,15]. Furthermore, a number of studies explored the pharmacological role of active constituents of various garlic preparations, such as aged garlic preparation, garlic oil, and garlic powder [16,17]. Since garlic is one of the most commonly used herbs and nutritional supplements used in different forms in almost every part of the world, there is a possibility that the use of this substance may interact with the conventional therapies that patients use for their chronic ailments. Therefore, we have previously reported garlic’s cardiovascular actions and interactions and its different preparations with several drugs. In our previous studies, garlic had an antihypertensive benefit and increased the bioavailability and antihypertensive effects of propranolol and hydrochlorthiazide [18,19,20]. Captopril’s cardioprotective and hypertension effects were enhanced by garlic and its active ingredient s-allyl cysteine (SAC) [21]. We also reported that garlic, aged garlic extract, and SAC had antioxidant and hypolipidemic properties that were modified by antihypertensive treatment [22]. We recently published a study examining how aged garlic extract and its ingredient SAC affected isoprenaline-induced myocardial infarction in rats [16].

Organosulfur chemicals, saponins, phenolic compounds, and polysaccharides are among the beneficial compounds found in garlic [23]. The major active elements in garlic oil are organosulfur compounds such as diallyl thiosulfonate (allicin), diallyl sulfide (DAS), diallyl disulfide (DADS), diallyl trisulfide (DATS), SAC, and S-allyl-cystein sulfoxide [24]. Garlic oil is a traditional garlic preparation that has been demonstrated to increase antioxidant enzyme activity, inhibit 1,3-dichloro-2-propanol metabolic activation, and reduce apoptosis in the liver, indicating a protective effect against liver injury in rats [25]. Garlic active components, including DAS, DADS, and S-methyl-l-cysteine, have also been shown to protect and treat liver damage, including acute and chronic ethanol-induced liver damage [26]. Garlic oil has been shown to help people lose weight by lowering low-density lipoprotein (LDL) cholesterol levels [27]. Since hyperlipidemia is a known trigger for MI, we intend to find out the role of garlic oil in attenuating ischemic damage and also to explore whether its major active constituent, DADS, possesses similar or different cardioprotective efficacy.

Beta-adrenergic blockers are a group of medicines that have a variety of pharmacological effects. Carvedilol is a nonselective beta-blocker that also inhibits alpha1-adrenergic receptors, resulting in cardioprotection and vasodilation. It is used to treat hypertension, angina pectoris, and cardiac arrhythmias, as well as to act as an antioxidant and antiproliferative substance. Left ventricular dysfunction and congestive heart failure are treated with carvedilol. Aside from these benefits, it also lowers cholesterol and improves renal impairment, implying widespread usage of this nonselective beta-blocker [28].

Continuing our efforts to determine the beneficial effects of garlic preparations and their influence on the effects of drugs affecting cardiovascular functions, the present study determined the effect of garlic oil and diallyl disulfide (DADS) on the cardioprotective effect of carvedilol on chronic isoprenaline injection mediated myocardial necrosis in rats.

## 2. Results

### 2.1. Effect on Heart Weight and Heart to Body Weight Ratio

Table 1 exhibits the heart weight and the ratio of heart weight to body weight at the end of 14 days of treatment. A significant (*p* < 0.001) increase in heart weight and heart weight to body weight ratio was noticed in animals which received only chronic doses of isoprenaline (isoprenaline control) when compared to the normal control group. However, all animals pretreated with garlic oil, diallyl disulfide, and carvedilol showed significantly (*p* < 0.01) decreased heart weight and heart to body weight ratio compared to isoprenaline control. Additionally, animals that were given either garlic oil/diallyl disulfide along with carvedilol demonstrated significant (*p* < 0.05) improvement in their heart weight and heart weight to body weight ratio in comparison with their garlic oil and diallyl disulfide groups, respectively.

### 2.2. Effect on Serum Cardiac Marker Enzymes

A significant (*p* < 0.001) increase in cardiac marker activities was observed in the isoprenaline control group when compared to normal control, signifying potential damage to the cardiac musculature (Table 2). The group of animals that were treated with garlic oil, diallyl disulfide, and carvedilol showed a significantly (*p* < 0.001) decreased cardiac markers in the serum when compared to isoprenaline control group animals. Furthermore, animals that received both GO or DADS and carvedilol exhibited further significant declines in CK-MB (*p* < 0.01), LDH (*p* < 0.01), and cTnC (*p* < 0.05) activities when compared to groups of animals that received only GO or DADS, respectively.

### 2.3. Effect on Antioxidants and Lipid Peroxidation Product

As evident from Table 3, both cardiac antioxidant enzymes, SOD, and CAT activities were significantly (*p* < 0.001) depleted with a simultaneous elevation in TBARS level in isoprenaline injected rats compared to in normal control rats. On the contrary, all treated animals showed a significant (*p* < 0.001) incline in SOD, CAT activities and a decline in TBARS levels compared to isoprenaline control rats. It was also seen that the animals that concurrently received GO or DADS with CAR exhibited significantly (*p* < 0.05) more SOD, CAT activities, and fewer TBARS activities in their heart tissue homogenate than the isoprenaline injected rats.

### 2.4. Effect on Cytosolic and Nuclear Inflammatory Markers

As evident from Table 4, the concentration of both cytosolic and nuclear inflammatory markers was significantly (p < 0.001) increased in animals given chronic doses of isoprenaline compared to in those that received only the normal vehicle. On the other hand, those animals that received treatment with GO, DADS, or CAR had a significantly (*p* < 0.001) lower concentration of inflammatory markers when compared to the isoprenaline control group. Importantly, those rats that received both GO and CAR, or DADS and CAR, demonstrated a significantly (*p* < 0.05) decreased concentration of the inflammatory markers compared to those that received only GO or DADS alone.

## 3. Discussion

The results obtained from this study demonstrate the potent cardioprotective potential of garlic oil (GO), its active constituent, diallyl disulfide (DADS), and carvedilol by improvement of antioxidant status and alleviating inflammation by downregulating nuclear factor kappa b signaling mechanism. Furthermore, this study also exhibited the enhanced cardioprotective benefits of combining GO or DADS with carvedilol during isoprenaline-mediated damage to the cardiac musculature of rats.

Isoprenaline (ISO) administration triggers a series of events that eventually lead to cardiac hypertrophy with disruption and rupturing of the cardiac musculature that results in movement of water from the extracellular fluid and an increase in heart weight [29,30]. In accordance with this, the weight of the heart, as well as the ratio between the heart weight and the body weight, were remarkably high in isoprenaline injected rats, which was successfully alleviated in rats that received 14 days of garlic oil, diallyl disulfide, or carvedilol treatment either separately or together. This indicates that our treatments considerably protected the cardiac musculature from ISO-mediated rupture by decreasing oxidative stress and decreasing the movement of water to the cardiomyocytes, thereby maintaining the homeostasis of the cardiac system. Agents that possess antioxidant potential are known to provide protection to the myocardium and prevent disruption of homeostasis [31]. In addition to garlic oil and DADS, carvedilol also displayed antioxidant actions, although the relevance of this property remains uncertain [32].

The cardiac musculature is highly sensitive to oxidative free radical-induced damage as it is rich in polyunsaturated fatty acids (PUFA) with reduced cardiac antioxidant potential [33]. During times of cardiac stress, such as isoprenaline-induced oxidative stress, the activities of cardiac enzymes fall, exposing myocytes to further damage. However, both garlic oil and DADS, as well as even carvedilol, caused substantial improvement in the activities of both cardiac antioxidants, CAT and SOD, with a reduction in lipid peroxidation. Our findings are in accordance with an earlier report on the antioxidant potential of garlic oil and its organosulfur compounds, including DADS [34] and diallyl trisulfide (DATS) [35]. The antioxidant action of garlic oil is obviously due to organosulphurous compounds, but the exact mechanism is still not clear. A previous study employing DATS, another potent component of garlic, suggested cardioprotective effectiveness by lowering AGE-induced cardiomyocyte apoptosis by removing reactive oxygen species (ROS) and downstream PKCδ signaling [36]. Another interesting study explored the role of this polysulfide (DATS) and reported its effective mitigation of metabolic syndrome (MetS) as well as protective effects against ex vivo induced myocardial ischemia-reperfusion injury in MetS rats [37]. It is apparent that allyl sulfur constituents of these preparations are responsible for accelerated antioxidant enzyme synthesis at times of isoprenaline-induced stress to the myocardium [38]. Furthermore, the free radical production that is triggered by isoprenaline administration attacks the PUFA of the cardiac myocytes and causes their rupture, resulting in the release of enzymes from myocytes into the bloodstream [39]. This results in an elevated level of cardiac markers such as LDH, CK-MB, and cTnC in the blood. Therefore, chronic administration of isoprenaline caused a significant elevation in these cardiac marker enzymes. However, the protection offered by garlic oil, DADS, and CAR ameliorated this deleterious effect to a considerable extent, and the level of these markers are close to the normal control values even with chronic ISO administration [40]. 

It is also important to emphasize that both inflammation and oxidative stress play a crucial role in developing isoprenaline-induced myocardial infarction. Oxidative stress is viewed as an imbalance between the production of reactive oxygen species (ROS) and their elimination by protective mechanisms, which can lead to chronic inflammation. Differential expression of several genes implicated in inflammatory pathways can be caused by oxidative stress activating a number of transcription factors. Thus, the concentration of inflammatory markers such as interleukin one beta (IL-1β), tumor necrosis factor alpha (TNF-α), interleukin six (IL-6), and kappa bp65 subunit (NF-b p65) were assessed in the cytosolic fraction. The inflammatory markers concentrations increased enormously in animals that received isoprenaline, while garlic oil and its active constituent, DADS, as well as carvedilol, significantly depleted the concentration of these inflammatory markers. The outcome of this study on inflammatory markers is similar to a study done by Zare et al., 2019 [41]. Our study used garlic oil, while the earlier study prepared garlic extract to determine the inflammatory markers of peritoneal dialysis patients. Nevertheless, this study is the first attempt to determine the inflammatory marker concentration in myocardial infarction animals. It is possible that garlic oil and DADS downregulate the expression of inflammatory markers by inhibiting the NF-kappa b signaling pathway and exhibit cardioprotection against isoprenaline-induced myocardial dysfunction [42].

The function of garlic oil and DADS in enhancing the cardioprotective efficacy of carvedilol in ISO-induced ischemia damage in experimental mice is reaffirmed in this research study. Despite the fact that beta-blockers are well-known for their cardioprotective benefits, this research study was the first to discover carvedilol’s cardioprotective interaction with garlic oil and DADS in rats during myocardial stress. In addition, the significance of inflammation in causing myocardial stress was highlighted in this study. Both GO, and DADS were able to significantly reduce inflammatory markers in both the cytosolic and nuclear fractions. Finally, this study’s findings will open the way for more research into the role of garlic oil and DADS in the therapeutic regimen, potentially lowering the dosage of standard cardioprotective drugs such as carvedilol.

## 4. Materials and Methods

### 4.1. Experimental Animals

Sprague-Dawley rats (12–16 weeks old) weighing 150–200 g were kept at 25 ± 5 °C in a well-ventilated animal housing with a 12:12 h light–dark cycle. The rats had unrestricted access to standard rat chow, which contained 22.10 percent protein, 4.13 percent oil, 3.15 percent fiber, 5.15 percent ash, 1.12 percent sand (silica), and 1.12 percent and water ad libitum. All necessary steps and precautions were placed as per the ethical requirements of the Bioethics committee of King Abdulaziz City for Science and Technology (KACST), Saudi Arabia (KACST). The research committee of the College of Pharmacy, AlMaarefa University provided approval for this research project [MCST (AU)-COP 1934/RC].

### 4.2. Materials

Sigma Aldrich (USA) provided the garlic oil, which was GC-MS standardized for the presence of DADS. All of the compounds utilized in this investigation were analytical grade and came from a standard source. The CK-MB and LDH kits were provided by Crest Biosystems and Coral Clinical Systems (Goa, India). The DL-isoproterenol hydrochloride was provided by Sigma Aldrich (St. Louis, MO, USA). All chemicals for the project were purchased from Al-Majharia international trading establishment in Saudi Arabia, while glassware and other required equipment were provided by Gulf Scientific Glass Industry limited in Saudi Arabia and Scientific equipment trading Establishment in Saudi Arabia, respectively.

### 4.3. Experimental Grouping

At the end of one week of the acclimatization period, all 42 rats were randomly divided into seven groups, with six in each group. Group I (normal control) and II (isoprenaline control) were given saline (1 mL/kg, p.o) for 14 days. Group III, IV and V were administered with garlic oil (GO, 100 mg/kg) [43], diallyl disulphide (DADS, 8.94 mg/kg) and carvedilol (CAR, 2 mg/kg) [44], respectively, for 14 days. The DADS dose was determined to be 8.94 mg/kg (equivalent to 100 mg/kg of garlic oil) based on GC-MS peaks of garlic oil in our laboratory experiment. Animals of groups VI and VII received GO + CAR and DADS + CAR, respectively, for 14 days. Animals were administered with respective treatment via gastric intubation tube. During the last 10 days of treatments, all rats of group II to group VII also were subcutaneously injected isoprenaline (ISO) 3 mg/kg/day [8]. 

### 4.4. Measurement of Parameters

At the end of the treatment period, rats were weighed, and blood was withdrawn under anesthesia induced by a combination of ketamine hydrochloride (75 mg/kg, i.p) and xylazine (10 mg/kg, i.p) [45]. The collected blood was centrifuged at 2500 rpm for 20 min at 4 °C, and serum samples were separated and stored at −80°C until analysis. After that, the animals were sacrificed, and the hearts were removed and separated from the surrounding veins and fatty tissue mass using scissors. Following that, the hearts were sliced open, flushed with salt water (0.9 percent NaCl), and dried. The hearts’ weights were measured [46]. Using a mortar and pestle, the heart homogenate was then set up in an ice-cold 0.25 M sucrose solution. The homogenate was then centrifuged for 15 min at 5000 rpm. The supernatant was drained, and biochemical and molecular analyses were carried out [12]. Heart tissue homogenate was used to measure superoxide dismutase (SOD) [47], catalase (CAT) [48], and thiobarbituric acid reactive substances (TBARS) [49]. To verify heart function or integrity, the activities of serum cardiac marker enzymes such as cardiac troponin C (cTnC), lactate dehydrogenase (LDH), and creatine kinase-MB (CK-MB isoform) were measured. Commercially available kits were used to estimate all three markers. The cytosolic and nuclear fractions of the cardiac tissue homogenate were separated using the nuclear/cytosolic fractionation kit. Inflammatory markers such as tumor necrosis factor alpha (TNF-α), interleukin one beta (IL-1β), and interleukin six (IL-6) were assessed in the cytosolic portion, whereas in the nuclear portion, kappa bp65 subunit (NF-b p65), was measured [50]. All calculations were made with ELISA kits from a local standard company’s supplier. The assessments are performed in accordance with the kit’s specifications.

### 4.5. Data Analysis

For each group of six rats, all values are reported as mean ± standard error of the mean (SEM). The *p*-value was calculated using a 0.05 significance level and a comparison of normal control vs. ISO control, ISO control vs. all treatments, GO/DADS vs. GO + CAR/DADS + CAR. To establish statistical significance, a one-way analysis of variance (ANOVA) was utilized, followed by Dunnet comparison tests using the GraphPad Prism 8.0 (San Diego, CA, USA) computer software kit.

## 5. Conclusions

Treatment of animals with GO, its active constituent, DADS, or CAR either individually or together, provided significant protection to the myocardium. The protection offered by GO or DADS is augmented by the presence of CAR whereas, both GO, and DADS exhibit almost similar types of cardioprotective potential. The treatments offered protection both by ameliorating oxidative stress and by downregulating the inflammatory signaling pathway. However, further experiments are needed to determine the mechanism of the interaction between garlic and DADS with carvedilol.

## Figures and Tables

**Table 1:** Effect on heart weight and heart/body weight ratio.

Groups	Heart Weight (g)	Body Weight (g)	Heart to Body Weight Ratio (Percentage)
Normal control	0.437 ± 0.08	175.23 ± 4.32	0.249 ± 0.01
Isoprenaline control	0.670 ± 0.05 ***	172.32 ± 5.87	0.388 ± 0.01 ***
GO	0.514 ± 0.06 ^●●^	171.45 ± 5.22	0.299 ± 0.01 ^●●^
DADS	0.521 ± 0.07 ^●●^	174.21 ± 6.37	0.299 ± 0.01 ^●●^
CAR	0.482 ± 0.06 ^●●^	173.56 ± 2.38	0.277 ± 0.01 ^●●●^
GO + CAR	0.446 ± 0.08 ^●●●▫^	175.89 ± 3.69	0.253 ± 0.01 ^●●●▫^
DADS + CAR	0.431 ± 0.04 ^●●●▫^	173.37 ± 5.55	0.248 ± 0.01 ^●●●▫^

Values are given as mean ± standard error of mean for 6 rats in each group; *** *p* < 0.001 when compared to normal control; ^●●^ *p* < 0.01, ^●●●^ *p* < 0.001 compared to Isoprenaline control; ^▫^ *p* < 0.05, compared to GO/DADS respective groups; DADS, diallyl disulfide; GO, garlic oil; CAR, carvedilol.

**Table 2:** Effect on serum cardiac markers.

Groups	LDH (unit/L)	CK-MB (unit/L)	cTnC (ng/mL)
Normal control	643.21 ± 21.11	643.11 ± 5.43	0.54 ± 0.11
Isoprenaline control	1248.37 ± 11 ***	1165.21 ± 5.44 ***	1.388 ± 0.12 ***
GO	944.54 ± 22.90 ^●●●^	825.95 ± 17.27 ^●●●^	0.72 ± 0.08 ^●●^
DADS	932.33 ± 17.90 ^●●●^	916.88 ± 19.14 ^●●●^	0.71 ± 0.09 ^●●^
CAR	953.21 ± 21.30 ^●●●^	933.31 ± 18.81 ^●●●^	0.68 ± 0.02 ^●●●^
GO + CAR	765.2 ± 4.86 ^●●●▫▫^	788.6 ± 12.73 ^●●●▫▫^	0.53 ± 0.11 ^●●●▫^
DADS + CAR	737.81 ± 2.38 ^●●●▫▫^	715.4 ± 11.31 ^●●●▫▫^	0.55 ± 0.08 ^●●●▫^

Values are given as mean ± standard error of mean for 6 rats in each group; *** *p* < 0.001 when compared to normal control; ^●●^ *p* < 0.01, ^●●●^ *p* <0.001 compared to Isoprenaline control; ^▫^ *p* < 0.05, ^▫▫^ *p* < 0.01 compared to GO/DADS respective groups; DADS, diallyl disulfide; GO, garlic oil; CAR, carvedilol.

**Table 3:** Effect on cardiac antioxidants and lipid peroxidation product.

Groups	SOD (units/mg Protein)	Catalase (units/mg Protein)	TBARS (units/mg Protein)
Normal control	12.46 ± 1.65	14.22 ± 1.16	21.32 ± 2.45
Isoprenaline control	3.99 ± 0.29 ***	5.46 ± 1.59 ***	41.06 ± 2.33 ***
GO	6.92 ± 1.80 ^●●●^	8.50 ± 4.01 ^●●●^	27.01 ± 4.15 ^●●●^
DADS	7.21 ± 2.71 ^●●●^	9.49 ± 2.54 ^●●●^	24.55 ± 3.57 ^●●●^
CAR	6.86 ± 1.05 ^●●●^	11.50 ± 2.28 ^●●●^	28.43 ± 2.81 ^●●●^
GO + CAR	9.21 ± 1.69 ^●●●▫^	13.41 ± 4.21 ^●●●▫▫^	22.83 ± 2.15 ^●●●▫^
DADS + CAR	10.28 ± 0.74 ^●●●▫^	14.82 ± 4.73 ^●●●▫▫^	18.74 ± 3.66 ^●●●▫^

Values are given as mean ± standard error of mean for 6 rats in each group; *** *p* < 0.001 when compared to normal control; ^●●●^ *p* < 0.001 compared to Isoprenaline control; ^▫^ *p* < 0.05, ^▫▫^ *p* < 0.01 compared to GO/DADS respective groups; DADS, diallyl disulfide; GO, garlic oil; CAR, carvedilol.

**Table 4:** Effect on inflammatory markers.

Groups	IL-1β (ng/mg)	TNF-α (ng/mg)	IL-6 (ng/mg)	NF-b p65 (ng/mg)
Normal control	58.61 ± 6.65	112.69 ± 2.54	65.32 ± 2.45	72.32 ± 7.55
Isoprenaline control	124.76 ± 3.87 ***	213.22 ± 2.28 ***	134.06 ± 4.32 ***	152.06 ± 7.45 ***
GO	78.87 ± 2.88 ^●●●^	165.35 ± 4.83 ^●●●^	87.01 ± 4.56 ^●●●^	112.01 ± 5.43 ^●●●^
DADS	81.20 ± 3.99 ^●●●^	159.43 ± 4.72 ^●●●^	79.55 ± 6.76 ^●●●^	109.55 ± 4.57 ^●●●^
CAR	92.76 ± 4.15 ^●●●^	171.76 ± 4.44 ^●●●^	92.43 ± 6.77 ^●●●^	115.43 ± 4.84 ^●●●^
GO + CAR	63.21 ± 5.49 ^●●●▫^	122.20 ± 4.93 ^●●●▫^	72.83 ± 7.44 ^●●●▫^	82.83 ± 24.15 ^●●●▫^
DADS + CAR	61.20 ± 3.84 ^●●●▫^	118.93 ± 2.74 ^●●●▫^	68.74 ± 6.66 ^●●●▫^	76.74 ± 5.32 ^●●●▫^

Values are given as mean ± standard error of mean for 6 rats in each group; *** *p*< 0.001 when compared to normal control; ^●●●^ *p* < 0.001 compared to Isoprenaline control; ^▫^ *p* < 0.05 compared to GO/DADS respective groups; DADS, diallyl disulfide; GO, garlic oil; CAR, carvedilol.; IL-1β, interleukin one beta; TNF-α, tumor necrosis factor alpha; IL-6, interleukin six; NF-b p65, nuclear factor kappa bp65 subunit.

## Data Availability

Data is contained within the article.

## Sample Availability

Samples of the compounds are available from the authors.
